# Germany-wide citizen science study reveals spread of *Babesia canis*-infected *Dermacentor reticulatus* ticks by dogs travelling within the country

**DOI:** 10.1016/j.crpvbd.2024.100187

**Published:** 2024-06-11

**Authors:** Andrea Springer, Alexander Lindau, Julia Probst, Katrin Fachet, Ingo Schäfer, Gerhard Dobler, Ute Mackenstedt, Christina Strube

**Affiliations:** a Institute for Parasitology, Centre for Infection Medicine, University of Veterinary Medicine Hannover, Buenteweg 17, 30559, Hanover, Germany; b Institute of Biology, Department of Parasitology, University of Hohenheim, Emil-Wolff-Straße 34, 70599, Stuttgart, Germany; c LABOKLIN GmbH and Co. KG, Bad Kissingen, Steubenstraße 4, 97688, Bad Kissingen, Germany; d Bundeswehr Institute of Microbiology, Neuherbergstr. 11, 80937, Munich, Germany; e Division of Infectious Diseases and Tropical Medicine, Medical Center of the University of Munich (LMU), Munich, Germany

**Keywords:** Ornate dog tick, Meadow tick, Canine babesiosis, Piroplasmosis, Tick-borne diseases, Endemic foci

## Abstract

The ornate dog tick *Dermacentor reticulatus*, vector of *Babesia canis*, has shown a considerable range expansion in several European countries. Previously, only few areas in Germany were recognised as endemic for *B. canis*, but a marked increase in autochthonous canine babesiosis cases and spread to new areas has been noted recently. To better assess the current risk for dogs, the present study screened 5913 specimens of *D. reticulatus* from all over Germany, collected in the frame of a Citizen Science study during 2019–2023. Moreover, 343 *Dermacentor marginatus* ticks were also included. *Babesia* detection was achieved by quantitative real-time PCR (qPCR). Positive samples were confirmed by sequencing. Moreover, a MGB-probe-based triplex qPCR was established to detect and distinguish between the canine *Babesia* spp. relevant in Europe, i.e. *B. canis*, *Babesia vogeli* and *Babesia gibsoni*. Overall, *B. canis* DNA was detected in five *D. reticulatus* specimens (0.08%). Two of the *B. canis*-positive ticks originated from areas previously known as endemic for canine babesiosis, namely from the area of Freiburg im Breisgau, federal state of Baden-Wuerttemberg, and from the district St. Wendel, federal state of Saarland. Three further *B. canis*-positive ticks were detected in districts not yet recognised as endemic, one each in the district of Mansfeld-Suedharz, federal state of Saxony-Anhalt, the district of Ravensburg, federal state of Baden-Wuerttemberg and in the city of Fürth, federal state of Bavaria. However, the tick in Fürth was found on a dog who had returned from a trip to the Breisgau region on the previous day, indicating translocation of the specimen out of this well-known endemic focus. The geographical distribution of the positive samples shows that *B. canis* is currently spreading in Germany, particularly *via* dogs travelling within the country, increasing the infection risk throughout the country. Important measures to contain a further spread of the pathogen include comprehensive year-round tick prophylaxis with licensed acaricides, not only to protect the individual pet, but also the entire dog population. Moreover, screening of dogs entering Germany from *B. canis*-endemic countries is required and any treatment should aim at pathogen elimination by use of appropriate imidocarb dosages.

## Highlights

• Five of 5913 *Dermacentor reticulatus* from all over Germany were positive for *Babesia canis* DNA.
• Three positive ticks originated from districts not yet recognised as endemic.
• Translocation by a travelling dog from an endemic to a non-endemic area was indicated.
• Further spreading needs to be prevented by year-round tick prophylaxis.
• Prevention also includes testing of dogs imported from endemic countries.

## Graphical abstract

**Figure:**
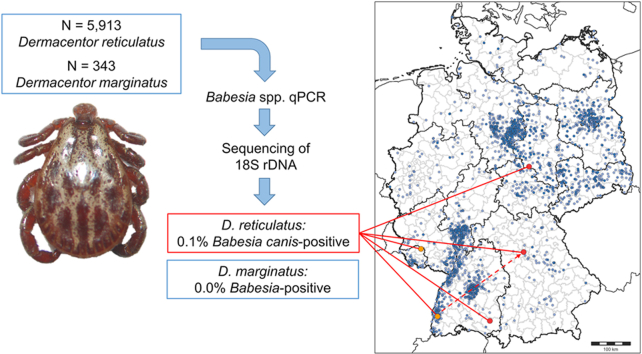

## 1 Introduction

Climatic and environmental changes may lead to an increased risk of infectious disease transmission, particularly with regard to vector-borne pathogens (Semenza and Suk, 2017; Nova et al., 2022). *Dermacentor reticulatus*, known as the ornate dog tick or meadow tick, has significantly expanded its range throughout central Europe during the 21st century (Bullová et al., 2009; Paulauskas et al., 2015; Mierzejewska et al., 2016; Daněk et al., 2022). In Germany, *D. reticulatus* has risen from a rarely encountered, focally distributed tick species during the 1960s/1970s (Negrobov and Borodin, 1964; Liebisch and Rahman, 1976) to the second most frequent tick species parasitising dogs (Probst et al., 2023a) within merely half a century. Its presence has now been recorded in all 16 German federal states (Springer et al., 2022). In contrast, a comparable spread has not been observed for the congeneric sheep tick *Dermacentor marginatus*, whose range is mainly restricted to southwestern Germany (Drehmann et al., 2020; Springer et al., 2022).

In addition to their vector function for zoonotic pathogens like *Rickettsia* spp. (Parola et al., 2009; Földvári et al., 2013) and – in the case of *D. reticulatus* – tick-borne encephalitis virus (Ličková et al., 2020), both *D. reticulatus* and *D. marginatus* are of veterinary relevance as vectors of piroplasmid parasites. While both species may transmit the causative agents of equine piroplasmosis, *Babesia caballi* and *Theileria equi* (Scoles and Ueti, 2015), *D. reticulatus* is of principal importance as vector for *Babesia canis* (Gray et al., 2019). These pathogens may cause severe to fatal disease in their vertebrate hosts, characterised by haemolytic anaemia, thrombocytopenia, systemic inflammatory reactions and subsequent multi-organ dysfunction, especially in immunologically naive animals (Wise et al., 2013; Solano-Gallego et al., 2016; Weingart et al., 2023).

**Fig. 1:**
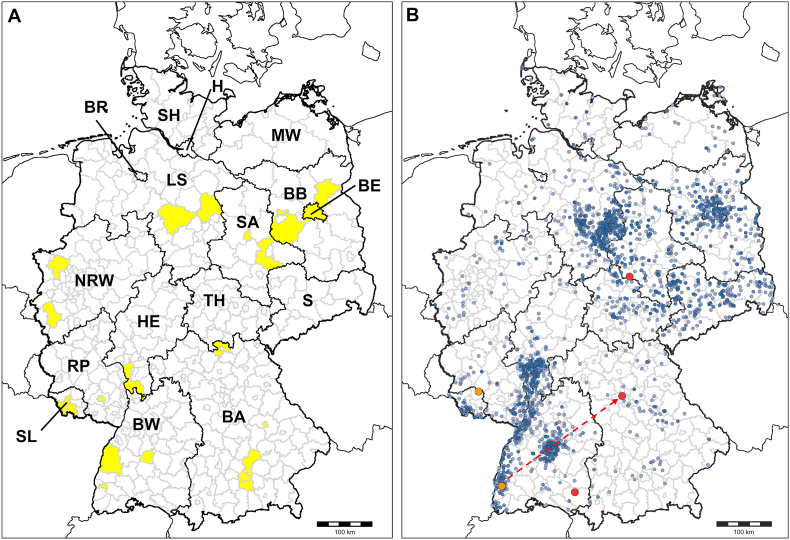
German districts with autochthonous canine babesiosis cases and/or detection of *Babesia canis* in ticks (highlighted in *yellow*) according to the literature (**A**) and geographical origin of *Dermacentor reticulatus* specimens tested in the present study (*blue dots*) (**B**), with *B. canis*-positive ticks in previously recognised endemic areas shown in orange and in new areas shown in red. The dashed arrow indicates probable translocation of the tick by a travelling dog. References for panel **A** are listed in Table 1. Federal states are abbreviated as follows: BA, Bavaria; BB, Brandenburg; BE, Berlin; BR, Free Hanseatic city of Bremen; BW, Baden-Wuerttemberg; H, Free and Hanseatic city of Hamburg; HE, Hesse; LS, Lower Saxony; MW, Mecklenburg-Western Pomerania; NRW, North Rhine-Westphalia; RP, Rhineland-Palatinate; S, Saxony; SA, Saxony-Anhalt; SH, Schleswig-Holstein; SL, Saarland; TH, Thuringia.

While autochthonous equine piroplasmosis cases are rare in Germany (Dirks et al., 2021), endemic foci of canine babesiosis due to *B. canis* have been identified in certain regions of the country. The first clusters of autochthonous infections were described in the Breisgau/Ortenaukreis region of the federal state Baden-Wuerttemberg in the 1980s and 1990s (Gothe et al., 1989; Gothe and Wegerdt, 1991; Zahler and Gothe, 1997). Further *B. canis*-endemic foci have been identified in the federal states of Bavaria, Berlin/Brandenburg, Hesse and Saarland, in addition to sporadic cases in other parts of the country (Table 1, Fig. 1A). In recent years, an increasing number of autochthonous infections in dogs has been reported, e.g. 78 cases in the Rhine-Main area of the federal state Hesse between 2018 and 2020 (Seibert et al., 2022) and 49 cases in the federal states of Berlin/Brandenburg between 2015 and 2021 (Weingart et al., 2023). In addition, the pathogen seems to have spread to new areas, such as Magdeburg in the federal state of Saxony-Anhalt (Johanna Lippert, personal communication). A similar emergence of new endemic foci has been recorded in other European countries, such as the UK (Wright, 2018) and Poland (Pawełczyk et al., 2022).

**Table 1:** German areas with previous detection of *Babesia canis* in ticks or autochthonous cases in dogs as described in the literature.

Location	*B. canis* detection (no. of positive dogs/ticks)	Reference
**Federal state of Baden-Wuerttemberg**		
District of Ortenaukreis (Offenburg)	Dogs (*n* = 70)	Gothe et al. (1989)
District of Ortenaukreis (Offenburg/Lahr)	Dogs (*n* = 85)	Gothe and Wegerdt (1991)
District of Ortenaukreis/City of Freiburg im Breisgau^a^	Dogs (*n* = 7)	Zahler and Gothe (1997)
City of Freiburg im Breisgau	Dogs (*n* = 3)	Gothe and Wegerdt (1991)
District of Tübingen (forest of Schoenbuch)	*D. reticulatus* (*n* = 1)	Naucke (2007)
**Federal state of Bavaria**		
District of Coburg (Roedental/Einberg)	*D. reticulatus* (*n* = 1)	Naucke (2007)
Freising (Isarauen)	*D. reticulatus* (*n* = 1)	Silaghi et al. (2020)
City of Munich	Dogs (*n* = 12)	Zahler et al. (2000b)
City of Regensburg	Dogs (*n* = 7)	Zahler et al. (2000a)
**Federal state of Brandenburg**		
District of Barnim (Prenden forest)	*D. reticulatus* (*n* = 1)	Naucke (2007)
District of Potsdam-Mittelmark (Bensdorf)	*D. reticulatus* (*n* = 2)	Naucke (2007)
**City of Berlin/Federal state of Brandenburg**^a^	Dogs (*n* = 49)	Weingart et al. (2023)
**Federal state of Hesse**		
District of Bergstraβe (Viernheim)	*D. reticulatus* (*n* = 1)	Naucke (2007)
Rhine-Main area	Dogs (*n* = 78)	Seibert et al. (2022)
**Federal state of Lower Saxony**		
District of Gifhorn	Dogs (*n* = ?)^b^	Martin Pape (pers. comm.)
Region of Hanover	Dog (*n* = 1)	Jensen and Nolte (2005)
**Federal state of North Rhine-Westphalia**		
District of Dueren	Dogs (*n* = 21)	Dürbaum (1999)
District of Wesel	Dogs (*n* = ?)^b^	Heile et al. (2006)
**Federal state of Rhineland-Palatinate**		
City of Kaiserslautern	Dog (*n* = 1)	Kehl et al. (2005)
**Federal state of Saarland**		
Region of Saarbrücken/District of Saarlouis^a^	*D. reticulatus* (*n* = 10)	Beelitz et al. (2012)
**Federal state of Saxony-Anhalt**		
District of Anhalt-Bitterfeld (Aken)	*D. reticulatus* (*n* = 1)	Naucke (2007)
Magdeburg	Dogs (*n* = ?)^b^	Johanna Lippert (pers. comm.)

a No further specification of the dogsʼ origin.

b Unknown number.

Despite the increasing number of autochthonous cases in dogs, pathogen detection in the German vector population has only been successful in a few cases so far (Table 1). Between 2004 and 2007, seven *B. canis*-positive ticks were identified among more than 2000 *Dermacentor* specimens from a Citizen Science study (Naucke, 2007). More targeted sampling of *D. reticulatus* populations in areas with reported canine babesiosis cases revealed a prevalence of 2.5% in the Saarbrücken/Saarlouis region of the federal state Saarland (Beelitz et al., 2012) and of 0.3% in the district of Freising, federal state of Bavaria (Silaghi et al., 2020).

To better assess the current risk of *B. canis* exposure for dogs in Germany, *Dermacentor* specimens collected from all over Germany in the frame of a Citizen Science study (Drehmann et al., 2020; Springer et al., 2022) during the years 2019–2023 were subjected to *Babesia* detection by quantitative real-time PCR (qPCR) and *Babesia* species differentiation by conventional PCR. Moreover, a minor groove binder (MGB)-probe-based triplex qPCR was established based on published primer-probe sequences (Troskie et al., 2019) and a newly designed *B. canis* MGB-probe to distinguish between the canine *Babesia* spp. relevant in Europe, namely *B. canis*, *Babesia vogeli* and *Babesia gibsoni*.

## 2 Materials and methods

### 2.1 Collected ticks

A Citizen Science study to assess the distribution of *Dermacentor* spp. was initiated in 2019, asking citizens to submit *Dermacentor* spp. ticks or “ticks of unusual appearance”, together with information on the location, host association and circumstances of finding as previously described (Drehmann et al., 2020; Springer et al., 2022). The tick specimens were sent to the participating research institutions *via* mail. Received *Dermacentor* specimens were identified by morphological criteria (Arthur, 1963; Siuda, 1991; Estrada-Peña et al., 2017), categorized as engorged or unengorged based on a visual assessment of their body dimensions and stored at −80 °C until further processing.

### 2.2 Nucleic acid extraction

Ticks collected in 2019 were transferred into 400 μl of Minimum Essential Medium (Sigma Aldrich, Taufkirchen, Germany) and homogenised with three steel beads of 3 mm diameter in a bead mill (MM400, Retsch GmbH, Haan, Germany) at a frequency of 30 Hz for 10 min. Subsequently, 200 μl of the homogenate was purified using the Maxwell® 16 Tissue DNA Purification Kit in a Maxwell® 16 instrument (both Promega GmbH, Walldorf, Germany) following the manufacturerʼs instructions and eluted in 50 μl elution buffer. The eluted DNA was stored at −80 °C until further use.

From the remaining ticks, nucleic acid was extracted using the NucleoSpin® 96 RNA kit in combination with the NucleoSpin® RNA/DNA Buffer Set (both Macherey-Nagel GmbH & Co. KG, Dueren, Germany). First, whole ticks were homogenised in 300 μl RA1 buffer with addition of 3 μl β-mercaptoethanol and one 3 mm steel bead for 2 × 2 min at 30 Hz in a TissueLyser II instrument (Qiagen, Hilden, Germany). The entire lysate was mixed with 300 μl RA4 buffer and transferred to a NucleoSpin® RNA binding plate for vacuum processing according to the manufacturerʼs instructions. DNA was eluted in 100 μl DNA Elute buffer and stored at −20 °C.

### 2.3 *Babesia* screening by genus-specific qPCR and conventional PCR

Molecular assays used for *Babesia* detection and differentiation in this study are summarized in Table 2. For initial screening for *Babesia* spp., a genus-specific SYBR Green-based qPCR amplifiying a 150 bp part of the mitochondrial lsu5-lsu4 region was performed (Qurollo et al., 2017). The 25 μl reaction contained 10 μl DNA template, 12.5 μl iTaq™ Universal SYBR® Green Supermix (Bio-Rad Laboratories GmbH, Feldkirchen, Germany) and 0.5 μl of each primer (30 μM each). The thermoprofile consisted of 95 °C for 10 min, followed by 40 cycles of 95 °C for 15 s and 60 °C for 1 min, with subsequent melting temperature (T_m_) measurements between 55 °C and 95 °C. All qPCRs included a negative control and *B. canis* plasmid DNA as a positive control.

**Table 2:** Molecular assays used for *Babesia* spp. detection and differentiation in *Dermacentor* spp. ticks in the present study.

Assay type	Target	Primers (Reference)	Probes (Reference)
SYBR Green-based qPCR (genus-specific)	Mitochondrial lsu5-lsu4 region	B-lsu-F & B-lsu-R2 (Qurollo et al., 2017)	NA
Conventional PCR (genus-specific)	18S rDNA	BJ1 & BN2 (Casati et al., 2006)	NA
Semi-nested conventional PCR (genus-specific)	18S rDNA	BJ1& BN2 (Casati et al., 2006); PiroB (Armstrong et al., 1998)	NA
MGB probe-based triplex qPCR (species-specific)	18S rDNA	BDogF & BDogR (Troskie et al., 2019)	Bcanis_IPH (This study), BVogeliP & BGibsoniP (Troskie et al., 2019)

*Abbreviation*: NA, not applicable.

To confirm positive qPCR results and to achieve *Babesia* spp. differentiation, positive samples with a T_m_ of 73.0–76.6 °C were subjected to conventional PCR targeting a 425 bp fragment of the 18S rRNA gene with primers BJ1 and BN2 (Casati et al., 2006). The 25 μl reaction contained 5 μl of DNA template, 2.5 μl 10×buffer, 0.5 μl of dNTPs (10 mM), 0.5 μl of each primer (10 μM each) and 0.25 μl DreamTaq® polymerase (Fisher Scientific GmbH, Schwerte, Germany). The thermoprofile consisted of 95 °C for 3 min, 40 cycles of 94 °C for 30 s, 55 °C for 30 s, 72 °C for 1 min, and final elongation at 72 °C for 10 min. In case no band or multiple bands were visible, semi-nested re-amplification with primers BJ1 and PiroB (Armstrong et al., 1998) was performed with 1 μl of the PCR product from the first PCR as a template, using the same thermoprofile as in the first round.

After electrophoresis on 1.5% agarose gels stained with GelRed (Biotium Inc., Fremont, CA, USA), the obtained amplicons were custom Sanger-sequenced (Microsynth Seqlab Sequencing Laboratories, Göttingen, Germany), edited in Clone Manager (v. Professional 9, Sci Ed Software, Westminster, CO, USA), and compared to publicly available sequences using NCBI BLAST. In case the obtained sequence matched with *Dermacentor* spp. instead of *Babesia* spp., the 150 bp lsu5-lsu4 qPCR product was additionally sequenced, and, if this matched to *Babesia* spp., the 18S PCR was repeated with template volume increased to 10 μl. *Babesia* sequences generated during this study were submitted to the GenBank database.

In case of a positive *Babesia* spp. result, the sender of the respective tick was contacted to obtain further information on a possible travel history of the host.

### 2.4 Establishment of a MGB probe-based triplex qPCR for *B. canis*, *B. vogeli* and *B. gibsoni*

As the SYBR Green-based qPCR resulted in a high number of false-positive samples due to amplification of *Dermacentor* spp. DNA, a MGB-probe-based triplex qPCR was established with the aim to improve specificity and to allow distinction between *B. canis*, *B. vogeli* and *B. gibsoni*, which is especially relevant for clinical samples in Europe. Primer-probe combinations targeting the 18S rRNA gene of these species have been previously published by Troskie et al. (2019). In the present study, a new *B. canis* probe was designed due to observed cross-reactions with the original probe and *B. gibsoni*, *B. vogeli* as well as *Babesia divergens* DNA.

Plasmid standards for *B. canis*, *B. vogeli* and *B. gibsoni*, respectively, were constructed from diagnostic samples that tested positive for these pathogens. After amplification of a 460–540 bp region of the 18S rRNA gene with primers RLB-F2 and RLB-R2 (Gubbels et al., 1999), the amplicons were ligated into TOPO™ TA vectors and transformed into One Shot™ TOP10 Chemically Competent *E. coli* (both Invitrogen™, Fisher Scientific GmbH, Schwerte, Germany). After purification, dephosphorylation and linearization of the plasmid DNA as described by Laabs et al. (2012), the DNA concentration was measured using a NanoDrop^TM^ 1000 instrument (PEQLAB Biotechnologie, Erlangen, Germany) and dilutions of 10^0^–10^6^ plasmid copies per μl were prepared to be used as controls in the qPCR.

The triplex qPCR assay was performed in duplicates. Each 25 μl reaction contained 12.5 μl ABsolute Blue QPCR low ROX mix (Fisher Scientific GmbH, Schwerte, Germany), 0.2 μl of primers BDogF and BDogR (50 μM each) (Troskie et al., 2019) and 0.06 μl of each probe with the following concentrations: 10 μM for *B. canis* (Bcanis_IPH, newly designed in this study: FAM-CGG TTT GAC CAT TTG GTT GGT-MGB) and *B. vogeli* (BVogeliP: HEX-AGT TTG CCA TTC GTT TGG-MGB) (Troskie et al., 2019), 100 μM for *B. gibsoni* (BGibsoniP: CY5-CCC GAC TCG GCT ACT-MGB) (Troskie et al., 2019). As template, 1 μl of each plasmid standard dilution and 10 μl of tick DNA were used, respectively. The thermoprofile included a polymerase activation step of 15 min at 95 °C, followed by 40 cycles of 94 °C for 20 s, 66 °C for 1 min and 72 °C for 45 s.

## 3 Results

### 3.1 *Babesia* spp. prevalence and geographical origin of positive samples

Of the ticks collected in the frame of the Citizen Science study (Drehmann et al., 2020; Springer et al., 2022), a total of 5913 *D. reticulatus* specimens received between 2019 and 2023 were analysed. *Babesia canis* infection was confirmed in five *D. reticulatus* specimens (0.08%), while no other *Babesia* spp. were detected. The obtained 18S rDNA sequences (GenBank accession nos: PP658453-PP658457) showed 100% nucleotide identity to published *B. canis* sequences (e.g. MN078319; 100% query cover). Moreover, 343 *D. marginatus* specimens collected during 2019 were also tested but yielded a negative result in the qPCR.

The geographical origin of all tested *D. reticulatus* specimens and of the *B. canis*-positive ticks, all of which were detected on dogs, is shown in Fig. 1B. Two positive ticks were collected in areas previously known as endemic for canine babesiosis (Fig. 1A), namely from the area of Freiburg im Breisgau, at the western border of the federal state Baden-Wuerttemberg, and from the district St. Wendel, federal state of Saarland (Table 3). A further positive specimen was detected on a dog in the city of Fürth, federal state of Bavaria, which has not yet been recognised as an endemic area. However, the owners reported that their dog had accompanied them on a trip to the mountain range of Kaiserstuhl, located near Freiburg im Breisgau, from which they had returned one day prior to detecting the tick. The remaining two ticks were found in the district of Mansfeld-Suedharz, federal state of Saxony-Anhalt, and district of Ravensburg, federal state of Baden-Wuerttemberg, respectively, which are also not known as endemic for canine babesiosis. In both cases, the owners reported that their dog had not travelled during the last 14 days before the tick was detected. The dog from Ravensburg showed unspecific clinical signs (reduced general condition, coughing, sneezing) and was therefore presented at a veterinary practice, where the engorged *D. reticulatus* specimen was found during the clinical examination. The dog had a slightly elevated body temperature of 39.2 °C. According to the veterinarian, the symptoms disappeared after 2–3 days. No further diagnostic tests were carried out.

**Table 3:** Detailed information on *Babesia canis*-positive *Dermacentor reticulatus* specimens collected in the frame of a Citizen Science study in Germany.

Tick stage	Tick engorgement status	Host	Location of finding (federal state)	Month/year of finding	Further information	District-level detection frequency in *D. reticulatus* (positive/analysed ticks)
Adult female	Unengorged, attachment status unknown^a^	Dog	City of Freiburg i. Breisgau (Baden-Wuerttemberg)	October 2019	No travel history reported	2.9% (1/34)
Adult male	Unengorged, not attached	Dog	City of Fürth (Bavaria)	October 2020	Returned from a trip to the Kaiserstuhl near Freiburg i. Breisgau (Baden-Wurttemberg) on the day prior to tick detection	NA (only one tick received from this district)
Adult male	Unengorged, attachment status unknown^a^	Dog	Wallhausen, district Mansfeld-Suedharz (Saxony-Anhalt)	November 2020	No travel history reported	1.7% (1/59)
Adult male	Unengorged, not attached	Dog	Moosberg-Richweiler, district St. Wendel (Saarland)	April 2021	No travel history reported	NA (only one tick received from this district)
Adult female	Engorged	Dog	Bad Waldsee, district Ravensburg (Baden-Wuerttemberg)	September 2021	No travel history reported; dog showed unspecific clinical signs (reduced general condition, coughing, sneezing), which disappeared after 2–3 days	20.0% (1/5)

*Abbreviation*: NA, not applicable.

a Multiple attached and unattached ticks found on this host.

### 3.2 Performance of the triplex MGB-probe-based triplex qPCR

Of the five *B. canis* positive samples identified by sequencing, three were also positive in the MGB-probe-based triplex qPCR, with a mean Ct value of 24.2, 25.3 and 26.3 (i.e. 1.3 × 10^4^, 1.2 × 10^4^ and 6.2 × 10^3^ copies), respectively, as compared to Ct values of 19.6, 19.3 and 20.8 in the SYBR Green-based qPCR. The two remaining samples, which were negative by MGB-probe-based triplex qPCR, showed Ct values of 34.5 and 28.5 in the SYBR Green qPCR.

No cross-reactions with the *B. vogeli* and *B. gibsoni* probes were observed. Further, 57 samples that were positive in the SYBR Green qPCR but yielded either no amplicon in the 18S PCR or a sequence matching to *Dermacentor* sp., bacterial (e.g. *Fusobacterium* sp.) or fungal (e.g. *Cladosporium* sp.) DNA and were hence regarded as false positives, yielded a negative result in the MGB-probe-based triplex qPCR.

## 4 Discussion

In Germany, canine babesiosis was mainly regarded as an imported infection in the past, with the exception of few endemic foci (Dennig et al., 1980; Gothe and Wegerdt, 1991; Zahler et al., 2000a). The recent spread of *D. reticulatus* throughout Germany entails the risk of an increasing endemisation of *B. canis* (Springer et al., 2022). Indeed, the number of autochthonous canine babesiosis cases seems to be increasing (Seibert et al., 2022; Weingart et al., 2023). In the present study, *B. canis* DNA was detected in five of 5913 tested specimens of *D. reticulatus* from Germany. Two of these *B. canis*-positive ticks were collected in previously known endemic foci in the federal states of Baden-Wuerttemberg and Saarland, while the remaining three were detected in areas not yet recognised as endemic for canine babesiosis, illustrating the spread of the pathogen. For one of the positive ticks, the travel history of the dog indicated that it had most likely been acquired in the long-known endemic focus in the Breisgau region in Baden-Wuerttemberg, and then translocated to the dogʼs place of residence in the federal state of Bavaria. This illustrates the important role of travelling dogs in the spread of canine babesiosis, although it cannot be entirely excluded that the tick was actually acquired at the dogʼs place of residence. However, even in the latter case, the pathogen must have been introduced there previously through translocation of *B. canis*-infected ticks by travelling dogs or other (wild) animals. Additionally, subclinically infected imported dogs, e.g. *via* animal welfare organisations, may have introduced the pathogen. This scenario also applies to the two other dogs on which unengorged *B. canis*-infected ticks were found in areas not yet known to be endemic and for which the owners have not reported any travelling activity within the last 14 days. Thus, the risk of *B. canis* infection is not limited to the previously described endemic foci anymore but has to be considered throughout the country.

Clinical cases may occur year-round (Seibert et al., 2022), as activity of *D. reticulatus* peaks during autumn and spring with considerable activity during the winter months (Probst et al., 2023b). To contain further spread and to protect the individual pet, the use of year-round tick control with licensed acaricides needs to be strongly recommended to pet owners. Additional measures need to be directed against subclinically infected dogs, which may serve as a source of infection for ticks. This includes *Babesia* screening and treatment of positive dogs entering Germany from *B. canis*-endemic southern and eastern European countries and use of appropriate imidocarb dipropionate dosages to achieve pathogen elimination. Currently, imidocarb dipropionate is not licensed in Germany and needs to be imported from other European countries. In the package insert of these products, a dosage of 2.12 up to 6.06 mg/kg is recommended for therapy (e.g. MSD Animal Health, 2021; S P Veterinaria, 2020; Vet-Agro Multi-Trade Company Sp. z o.o, 2023). However, the approved dose in the USA is 6.6 mg/kg (US Food and Drug Administration, 2022) and elimination of the infection has been proven experimentally at a dose of 7.5 mg/kg (Penzhorn et al., 1995). Several case reports indicate that dogs may remain PCR-positive after receiving up to 6.0 mg/kg imdidocarb dipropionate, even after repeated injections (Seibert et al., 2022; Weingart et al., 2023). Thus, practitioners in Germany need to be educated with regard to the necessity of using higher doses than indicated in the package inserts and the use of PCR tests to monitor treatment efficacy.

As at least one of the *B. canis*-positive ticks was found feeding on its host, it cannot be entirely excluded that they became infected during the recent blood meal as the dogs may have been asymptomatic *B. canis* carriers. However, this seems rather unlikely as the majority of ticks were not yet engorged. The dog infested with an engorged female specimen showed an impaired general condition and slightly elevated body temperature at the time of tick detection. However, further clinical signs included coughing and sneezing, and the dogʼs condition improved after two to three days, indicating that the animal probably suffered from a respiratory infection rather than canine babesiosis, or was able to quickly control the infection due to early tick removal. For the remaining dogs harbouring unengorged positive ticks, no clinical signs were reported. Regardless of the dogsʼ infection status, the detection of *B. canis* in their ticks indicates the presence of the pathogen beyond previously known endemic foci.

The overall *B. canis* prevalence in the tested ticks amounted to 0.08%. For the interpretation of this result, it should be considered that the ticks were collected all over the country, not specifically in regions with (suspected) endemic foci. Moreover, ticks were sent to the research institutions by the participating citizens *via* mail. Although the ticks were frequently still alive when reaching the laboratory, room temperature transport might have affected DNA quality in cases of dead ticks, so the *Babesia* prevalence may have been underestimated. In previous studies from other European countries, prevalence values varying from 0 to 82.6% were reported (summarized by Zygner et al., 2023), although vast differences in sampling size and geographical extent of the studies as well as different molecular detection methods make direct comparisons difficult. Even in countries considered endemic for canine babesiosis, large regional differences in prevalence rates were recorded. For example, prevalence ranged from 0% *B. canis*-positive *D. reticulatus* in western to 14.7% in eastern Slovakia (Kubelová et al., 2011), and, similarly, from 0% in western to 5.9% in eastern Poland (Dwużnik-Szarek et al., 2022). In Germany, previous studies on *D. reticulatus* ticks collected from vegetation in endemic foci reported up to 2.5% positive ticks (Beelitz et al., 2012; Silaghi et al., 2020).

Despite the large sample size, no other *Babesia* spp. apart from *B. canis* were detected in the present study, neither in *D. reticulatus* nor in the 343 additionally tested *D. marginatus* specimens. Presence of *B. caballi* in *D. reticulatus* ticks has been recorded at a low prevalence in other non-endemic countries, e.g. in The Netherlands, Belgium and the United Kingdom (Jongejan et al., 2015; Sands et al., 2022). In contrast to the rising incidence of canine babesiosis, only few clinical cases of *B. caballi* infections have been reported in Germany so far (Adam et al., 2017; Dirks et al., 2021; Vogt et al., 2024). However, data from a diagnostic laboratory show that horses are regularly tested *B. caballi-*positive by serology and sometimes by PCR in Germany (Axt et al., 2024), presumably after travelling to or import from other countries, and might act as a source of infection for ticks. Therefore, the risk of endemisation exists. *Theileria equi* is even more frequently detected in German horses (Axt et al., 2024); however, the employed SYBR Green qPCR in the present study did not target *Theileria* spp., as the study was primarily designed as a survey for *B. canis*.

As the SYBR Green qPCR showed a high number of false positive samples due to amplification of *Dermacentor* spp. DNA, a MGB-probe-based qPCR was additionally evaluated. Detection of *B. canis* was combined in a triplex assay with detection of *B. vogeli* and *B. gibsoni*. *Babesia vogeli* and *B. gibsoni* are not expected to occur in *D. reticulatus* ticks as they are transmitted by *Rhipicephalus sanguineus* (s.l.) and *Haemaphysalis* spp. However, *B. canis*, *B. vogeli* and *B. gibsoni* are the most relevant species with regard to canine *Babesia* infections in Europe and the triplex qPCR was designed with clinical samples in mind. Although the assay was more specific than the lsu5-lsu4 SYBR Green qPCR, as no false positives due to amplification of *Dermacentor* spp. DNA were observed, the sensitivity was lower. Qurollo et al. (2017) already reported a higher sensitivity of their lsu5-lsu4 qPCR as compared to a qPCR based on 18S rDNA. This is probably due to the fact that the mitochondrial lsu5-lsu4 region is present at higher copy numbers within the *Babesia* cells than the nuclear 18S rDNA gene. However, Qurollo et al. (2017) evaluated specificity of the lsu5-lsu4 qPCR only by using DNA isolated from blood samples of various vertebrate species, not by testing DNA isolated from ticks. Therefore, optimization of qPCR detection protocols for screening of ticks is still necessary to improve specificity without compromising sensitivity of the lsu5-lsu4 SYBR Green qPCR by Qurollo et al. (2017), or to improve the sensitivity of the MGB-probe-based triplex qPCR evaluated in this study.

## 5 Conclusions

The geographical distribution of the *B. canis-*positive samples in the present study shows that the pathogen is currently spreading in Germany. In one case, the travel history of the infested dog indicated translocation of the positive tick from an endemic focus to a new area, highlighting the important role of dogs travelling within the country for the spread of canine babesiosis. In light of the winter activity of *D. reticulatus*, essential measures to contain this spread include consistent year-round tick prophylaxis in dogs with licensed acaricides, not only to protect the individual pet, but also the entire dog population. Screening of dogs entering Germany from endemic southern and eastern European countries is another key factor in preventing further spread, and any treatment should aim at pathogen elimination by use of appropriate imidocarb dipropionate dosages.

## Funding

The study was financially supported by Intervet Deutschland GmbH.

## Ethical approval

Not applicable.

## CRediT authorship contribution statement

**Andrea Springer:** Investigation, Visualization, Writing – original draft. **Alexander Lindau:** Investigation, Writing – review & editing. **Julia Probst:** Investigation, Writing – review & editing. **Katrin Fachet:** Investigation, Writing – review & editing. **Ingo Schäfer:** Resources, Writing – review & editing. **Gerhard Dobler:** Conceptualization, Writing – review & editing. **Ute Mackenstedt:** Conceptualization, Writing – review & editing. **Christina Strube:** Conceptualization, Funding acquisition, Supervision, Writing – review & editing.

## Declaration of competing interests

The authors declare the following financial interests/personal relationships which may be considered as potential competing interests: Christina Strube has repeatedly lectured for and acted as a consultant for diagnostic and (veterinary) pharmaceutical companies and has previous and ongoing research collaborations with various diagnostic and (veterinary) pharmaceutical companies; Ingo Schäfer is an employee of LABOKLIN GmbH and Co. KG. The other authors declare that they have no known competing financial interests or personal relationships that could have appeared to influence the work reported in this paper.

## Data Availability

The data supporting the conclusions of this article are included within the article. The sequences generated during this study have been submitted to the GenBank database under the accession numbers PP658453-PP658457.
